# Increased p38-MAPK is responsible for chemotherapy resistance in human gastric cancer cells

**DOI:** 10.1186/1471-2407-8-375

**Published:** 2008-12-18

**Authors:** Xianling Guo, Nannan Ma, Jin Wang, Jianrui Song, Xinxin Bu, Yue Cheng, Kai Sun, Haiyan Xiong, Guocheng Jiang, Baihe Zhang, Mengchao Wu, Lixin Wei

**Affiliations:** 1 Tumor Immunology and Gene Therapy Center, Eastern Hepatobiliary Surgery Hospital, the Second Military Medical University, Shanghai, PR China; 2 Hang Zhou Sanitarium of PLA, Zhejiang, PR China; 3 Department of Oncology, ChangZheng Hospital, the Second Military Medical University, Shanghai, PR China

## Abstract

**Background:**

Chemoresistance is one of the main obstacles to successful cancer therapy and is frequently associated with Multidrug resistance (MDR). Many different mechanisms have been suggested to explain the development of an MDR phenotype in cancer cells. One of the most studied mechanisms is the overexpression of P-glycoprotein (P-gp), which is a product of the *MDR1 *gene. Tumor cells often acquire the drug-resistance phenotype due to upregulation of the *MDR1 *gene. Overexpression of *MDR1 *gene has often been reported in primary gastric adenocarcinoma.

**Methods:**

This study investigated the role of p38-MAPK signal pathway in vincristine-resistant SGC7901/VCR cells. P-gp and MDR1 RNA were detected by Western blot analysis and RT-PCR amplification. Mitgen-activated protein kinases and function of P-gp were demonstrated by Western blot and FACS Aria cytometer analysis. Ap-1 activity and cell apoptosis were detected by Dual-Luciferase Reporter Assay and annexin V-PI dual staining.

**Results:**

The vincristine-resistant SGC7901/VCR cells with increased expression of the multidrug-resistance 1 (*MDR1*) gene were resistant to P-gp-related drug and P-gp-unrelated drugs. Constitutive increases of phosphorylated p38-MAPK and AP-1 activities were also found in the drug-resistant cells. Inhibition of p38-MAPK by SB202190 reduced activator protein-1 (AP-1) activity and *MDR1 *expression levels and increased the sensitivity of SGC7901/VCR cells to chemotherapy.

**Conclusion:**

Activation of the p38-MAPK pathway might be responsible for the modulation of P-glycoprotein-mediated and P-glycoprotein-unmediated multidrug resistance in the SGC7901/VCR cell line.

## Background

Multidrug resistance (MDR) is a serious problem in chemotherapy and is one of the main causes of poor outcome following cancer treatment. The MDR phenotype is often related to overexpression of drug-efflux pumps in cancer cells. P-glycoprotein (P-gp), a 170-kDa transmembrane glycoprotein encoded by the *MDR1 *gene, is one of the best characterized drug efflux pumps [1-3]. Overexpression of P-gp on the surface of tumor cells allows removal of cytotoxic drugs out of the cell in an energy-dependent manner, thereby reducing drug accumulation and increasing multidrug resistance. In addition, inhibition of the P-gp function or inhibition of its expression could prevent the P-gp-mediated MDR phenotype and improve the effectiveness of chemotherapy[4]. However, there is accumulating evidence that P-gp-associated MDR cells develop other pathways instigating chemoresistance to P-gp-unrelated drugs such as cisplatin and 5-FU [5-9].

Expression of P-gp has been reported to be regulated through transcriptional and post-transcriptional mechanisms and by various endogenous and environmental stimuli that evoke stress responses [10]. The transcriptional factor AP-1 has been shown to mediate P-gp expression [11]. Regulation of the AP-1 pathway is highly complex and activation of certain signal pathways seems to stimulate the transcriptional activity of AP-1 [12]. Simultaneous expression of P-gp and activation of several signal pathways has been found in some cancer cells. Moreover, these pathways have been reported to regulate the expression of P-gp in some multidrug-resistant cell lines [13-15], and blocking these pathways with their specific inhibitors has also been found to reduce P-gp expression [13,16]. These studies suggest that signal pathways play a positive role in the regulation of P-gp expression

In the present study, we assessed p38-MAPK phosphorylation and AP-1 activity in drug-resistant and drug-sensitive gastric cancer cells. Furthermore, the effect of the p38-MAPK inhibitor SB202190 on the *MDR1 *gene expression and AP-1 activity was also tested.

## Methods

### Cell Culture and reagents

Drug-sensitive human gastric cancer cell SGC7901 and the corresponding vincristine-resistant cell SGC7901/VCR were kindly provided by the Institute of Digestive Diseases (Fourth Military Medical University). All cells were cultivated in RPMI1640 medium (Gibco) supplemented with 10% heat-inactivated fetal calf serum in a CO_2 _incubator. To maintain the drug-resistance phenotype of SGC7901/VCR cells, vincristine (1.0 μg/ml) was also added to the medium. Cisplatin, 5-fluorouracil (5-FU) and epirubicin were purchased from QILU PHARMA (JiNan, Shandong, China). SB202190 was obtained from TOCRIS (Ballwin, MO, USA). The AP-1 luciferase report plasmid and the dominant-negative mutant p38 (DN-p38) plasmid were kind gifts from Dr Chuanshu Huang [17,18].

### Cell Viability Assay

A total of 4,000 SGC7901/VCR and SGC7901 cells were seeded in a 96-well plate. After 24 hours, cells were treated with different concentrations of 5-FU, cisplatin, or epirubicin. After 72 hours, the MTT assay was performed to evaluate cell viability.

### Luciferase assay

Cells were cultured in a twenty four-well plate until they reached 85–90% confluence. In all, total 0.8–1 μg plasmid DNA (DN-p38 plasmid mixed with AP-1 luciferase report plasmid) and 2.5 μl LipofectAMINE 2000 (Invitrogen, Carlsbad, CA, USA) mixed together were used to transfect each well in the absence of serum. After 4–6 h, the medium was replaced with 10% fetal calf serum RPMI1640. Approximately 36 h after the beginning of the transfection, cells were lysed and Luciferase assays were performed using the Dual Luciferase Reporter Assay System (Promega, WI, USA). A Renilla luciferase plasmid was also cotransfected in each experiment as an internal control for transfection efficiency. The relative luciferase activity reported here is the mean of three replicate experiments.

### RT-PCR Amplification

RNA was extracted from cells using Trizol (Invitrogen, Carlsbad, CA, USA). cDNA was synthesized using MMLV reverse transcriptase(Promega, WI, USA) and 2 μg total RNA and oligo dT_18_-primers. Two-microliter aliquots of cDNA were used for PCR amplification and primers were as follows: sense 5'-AAGCTTAGTACCAAAGAGGCTCTG-3' and antisense 5'-GGCTAGAAACAATAGTGAAAACAA-3' for MDR-1 [19]; sense 5'-TGACGGGGTCACCCACACTGTGCCCATCTA-3' and antisense 5'-CTAGAAGCATTGCGGTGGACGATGGAGGG-3') for β-actin [20]. PCR used 32 cycles of 30 seconds at 94°C, 45 seconds at 58°C, and 30 seconds at 72°C for MDR1 and β-actin. PCR products were separated by 2% agarose gel electrophoresis, and bands were visualized under ultraviolet (UV) radiation after staining with ethidium bromide. Gels were photographed and bands were analyzed by computerized densitometry.

### Western-blot analysis

Protein extracts were prepared using a nuclear extract kit (Active Motif, Carlsbad, CA, USA). Protein samples (30 μg) were separated by SDS-polyacrylamide gel electrophoresis (PAGE) and transferred to nitrocellulose membranes. Membranes were incubated at 4°C overnight with various primary antibodies against P-gp (Sigma, St Louis, MO, USA), β-actin, and phosphorylated or non-phosphorylated extracellular kinase receptor kinases (ERKs), Jun N-terminal kinases (JNKs) and p38 kinase (Cell Signaling Technology, Beverly, MA, USA). The resulting immunoblots were visualized with horseradish peroxidase-coupled goat anti-rabbit or anti-mouse immunoglobulin using an enhanced chemiluminescence (ECL) substrate system (Amersham Biosciences, Piscataway, NJ, USA).

### Apoptosis analysis with annexin V-PI dual staining

Cells (2 × 10^5 ^per well) were cultured in six-well plates to 70%–80% confluence. Cells were then treated with the indicated concentrations of chemotherapeutic agents, with or without SB202190 (10 μM), for 24 hours. Cells were collected and the annexin V-PI dual-staining assay was performed according to the manufacturer's instructions ((Nanjing Keygen Biotech, China). Collected cells were briefly washed with ice-cold phosphate-buffered saline (PBS) twice and resuspended in 300 μl 1 × binding buffer containing 5 μl Annexin V and 5 μl Propidium iodide (PI) for 30 minutes at room temperature in the dark. After incubation; the cells were analyzed using a FACS Aria cytometer (Becton Dickinson; San Jose, CA).

### FACS analysis for MAP-kinase

Cells were collected and resuspended in 0.5–1 ml PBS and formaldehyde was added to a final concentration of 2–4%. The cells were fixed for 10 minutes at 37°C and then chilled on ice for 1 minute. For permeabilization, pre-chilled cells were centrifuged and resuspended in 90% ice-cold methanol. Cells were then incubated for 30 minutes on ice. Permeabilized cells (1 × 10^6^) were mixed with 100 μl 0.5% bovine serum albumin (BSA)/PBS containing 2 μg/ml phospho-ERK, phospho-p38 MAPK, or phospho-JNK-specific antibody and incubated for 1 hour at room temperature. Cells were then washed with 0.1% BSA/PBS followed by incubation with goat anti-mouse immunoglobulin G (IgG) fluorescein isothiocyanate (FITC)-conjugated antibodies (50 μg/ml, KPL) for 30 minutes at room temperature. Cells were washed again with 0.1% BSA/PBS, then resuspended in 0.5 ml PBS and analyzed by flow cytometry.

### Accumulation and efflux of Rh123 was measured by flow cytometry

The measurement of Rh123 accumulation was performed. Briefly, cells (5 × 10^5 ^per sample) were incubated with 1 μg/mL of Rh123 in the dark at 37°C in 5% CO_2 _for 120 min. SB202190 was added to cultures at the same time as Rh123. Following Rh123 accumulation, cells were washed twice with ice-cold Hanks' Balanced Salt (HBSS) (without phenol red), placed in HBSS with 10% fetal bovine serum on ice. The green fluorescence of Rh123 was measured by flow cytometry. For determination of Rh123 efflux, cells were loaded for 120 min with Rh123 in the absence of SB202190, and then the medium was replaced with Rh123-free medium containing SB202190, or the vehicle. Following efflux intervals of 60 min, the medium was removed, and the cells were washed twice with ice-cold HBSS and prepared for flow cytometry as described earlier.

### Statistical analysis

Values were expressed as means ± standard deviation (S.D.). Differences were analyzed using the Student's *t*-test. A P-value of < 0.05 was considered significant.

## Results

### SGC7901/VCR cells are more resistant to chemotherapy than SGC7901 cells

**Figure 1:**
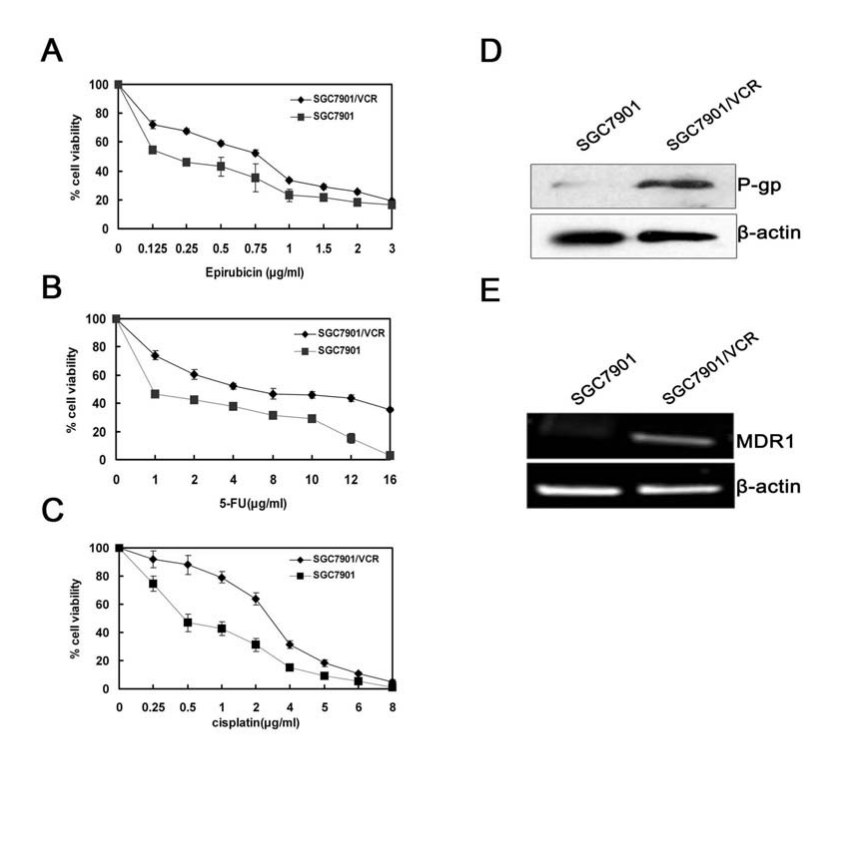
**SGC7901/VCR cells that overexpress *MDR1 *are more resistant to chemotherapy than SGC7901 cells**. (A-C) After treatment with different concentrations of 5-FU, cisplatin or epirubicin for 72 hours, cell viability was determined using the MTT assay. The viability of the untreated cells was taken as 100%. Points, mean of three independent experiments; bars, SD. Significant differences are indicated by asterisks. *, P < 0.05. (D) SGC7901/VCR and SGC7901 cells were harvested to prepare cell lysates. The lysates were subjected to SDS-PAGE and blotted with anti-P-gp antibody. (E) RT-PCR assays were performed to detect *MDR1 *mRNA. A representative example of an experiment that was repeated three times is shown.

The human gastric cancer cell line-SGC7901/VCR is a vincristine-resistant cell line that is derived from the vincristine-sensitive parent cell line SGC7901 [21]. To test whether SGC7901/VCR is more resistant to other chemotherapeutic agents than SGC7901, we treated these two kinds of cells with different concentrations of cisplatin, 5-FU and epirubicin. MTT assay results and IC_50 _values showed that SGC7901/VCR cells were more resistant to these chemotherapeutic agents. (Fig. 1A–C, Table 1 [see Additional file 1]). These results also indicated that SGC7901/VCR is a multidrug-resistant cancer cell line, which is resistant to P-gp-related drugs (vincristine and epirubicin) and P-gp-unrelated drugs (cisplatin and 5-FU).

### Increased expression of MDR1 in SGC7901/VCR cells

Although many different mechanisms have been suggested to explain the development of an MDR phenotype in cancer cells, one of the most extensively studied form of MDR is the P-gp-associated MDR phenotype, and a number of studies have convincingly supported that P-gp expression in tumor cells correlates with poor prognosis of chemotherapy [22]. Meanwhile, vincristine and epirubicin are known as P-glycoprotein substrate and cells resistant to these two drugs often overexpress P-glycoprotein. Therefore, we used Western-blot and RT-PCR analyses to investigate *MDR1 *gene expression in SGC7901 and SGC7901/VCR cells. As shown in Fig. 1D and 1E, SGC7901/VCR cells have increased expression levels of P-gp protein and MDR1 mRNA than SGC7901. Furthermore, we investigated whether cisplatin or 5-FU could induce *MDR1 *gene expression in SGC7901 cells; RT-PCR analyses showed that cisplatin or 5-FU treatment could not induce *MDR1 *gene mRNA expression (data not show). Thus, these results suggested P-gp-related or unrelated mechanism may involved in SGC7901/VCR cells to develop chemoresistance.

### Activation of p38-MAPK/AP-1 pathway in SGC7901/VCR cells

**Figure 2:**
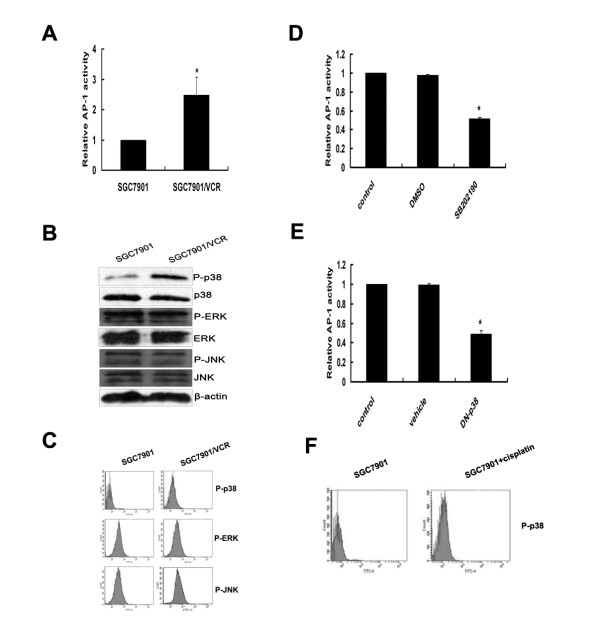
**Activation of p38 MAPK/AP-1 pathway in SGC7901/VCR cells**. (A) SGC7901/VCR and SGC7901 cells were transfected with AP-1 luciferase reporter plasmids. After 24 hours, the cells were collected and luciferase assays were performed. The luciferase activity results in SGC7901 cells were normalized to 1.0. Error bars indicate standard deviations. The values shown represent the means of at least three separate experiments. Significant differences are indicated by asterisks. *, P < 0.05. (B) Levels of phosphorylated and non-phosphroylated MAP kinases (p38-MAPK, ERKs and JNKs) in SGC7901/VCR and SGC7901 cells were detected using Western-blot analyses. A representative example of an experiment that was repeated three times is shown. (C) FACS analysis of p38-MAPK, ERK and JNK phosphorylation. A representative example of an experiment that was repeated three times is shown. (D-E) Analysis of AP-1 activity using the luciferase assay in SGC7901/VCR cells treated or untreated with SB202190 (10 μM), or cotransfected with the DN-p38 plasmid for 24 hours. The luciferase activity in control samples was normalized to 1.0. Error bars indicate standard deviations. The values shown represent the means of at least three separate experiments. Significant differences are indicated by asterisks. *, P < 0.05. (F) SGC7901 cells were incubated with cisplatin (2 μg/ml) for 24 h, and then cells were collected. FACS analysis was used to detect p38-MAPK phosphorylation.

Several studies have suggested that expression of *MDR1 *is regulated at the transcriptional level by multiple factors that can be activated via the stress-response pathways [23-26]. AP-1 is a well-known mediator of the stress-response pathways, which are composed of heterodimers from the Jun and Fos protein families [27]. Previous report has demonstrated that AP-1 is a transcription factor of *MDR1 *[11]. We therefore investigated AP-1 activities in SGC7901 and SGC7901/VCR cells using a luciferase reporter assay. Our results clearly showed that AP-1 activity was much greater in SGC7901/VCR cells than in SGC7901 cells (Fig. 2A).

AP-1 is a downstream target of the mitogen-activated protein (MAP) kinase pathway [27], and significant changes of MAP kinase levels in some drug-resistant cells have been reported [28,29]. We investigated the signaling pathway that regulates the activation of AP-1 in SGC7901/VCR and SGC7901 cells. Western-blot analysis of MAP kinases revealed that phosphorylation of p38-MAPK, but not ERKs and JNKs, is specifically increased in SGC7901/VCR cells (Fig. 2B). These results were confirmed by FACS analysis: SGC7901/VCR cells showed significant increased phosphorylation of p38-MAPK (Fig. 2C). Luciferase assay results also showed that inhibition of p38-MAPK by SB202190 or after transfection with the DN-p38 plasmid significantly reduced AP-1 activity in SGC7901/VCR cells (Fig. 2D, E). Furthermore, incubation with cisplatin for 24 h can also induce p38 phosphorylation in SGC7901 cell s (Fig. 2F). Taken together, these results showed that the p38-MAPK/AP-1 pathway was activated in SGC7901/VCR cells, and contributed to chemoresistance.

### Inhibition of p38-MAPK increases sensitivity of SGC7901/VCR cells to chemotherapy

**Figure 3:**
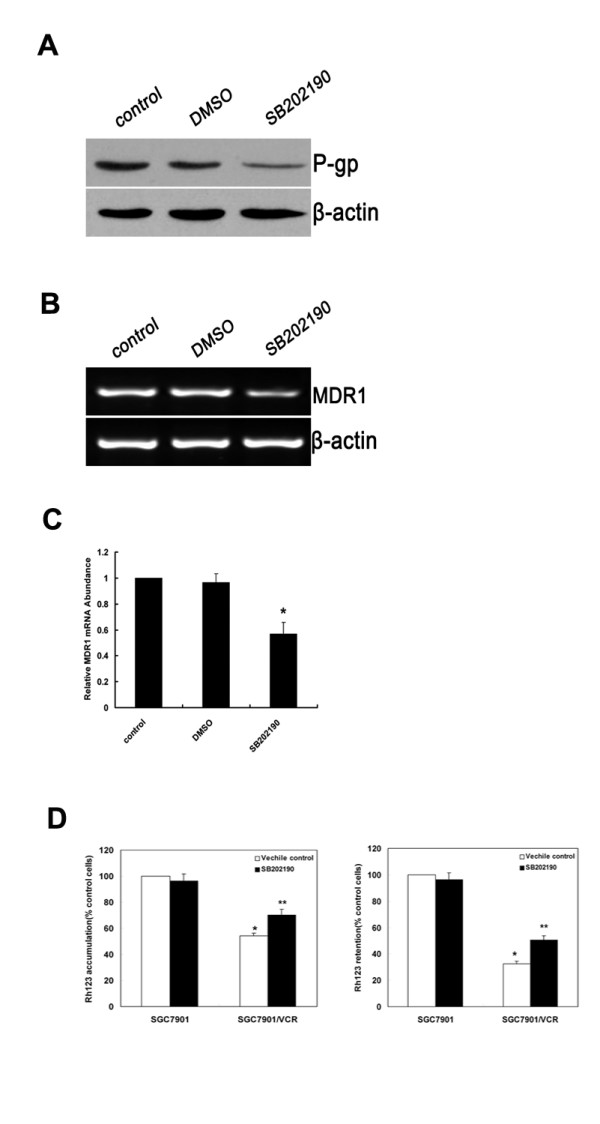
**Inhibition of p38-MAPK impairs MDR1 expression and function of P-gp in SGC7901/VCR cells**. SGC7901/VCR cells were treated with DMSO or SB202190 (10 μM). (A) Protein levels of P-gp were detected by Western-blot analysis. A representative example of an experiment that was repeated three times is shown. (B) SGC7901/VCR cells were treated with DMSO and SB202190. Expression of *MDR1 *mRNA was assessed by RT-PCR. β-actin mRNA levels were measured as positive internal controls. (C) The *MDR1 *mRNA expression levels were normalized to those of β-actin and are the means ± SD of at least three independent experiments. Significant differences are indicated by asterisks. *, P < 0.05. (D)Effects of SB202190 on Rh123 accumulation (left) and retention (right) in SGC7901 and SGC7901/VCR cells. (left) Cells treated with SB202190 (10 μM) or vehicle control (0.1% DMSO). Rh123 (1 μg/mL) was added, and the cells were incubated for 120 min. (right) Cells were incubated with Rh123 for 120 min, washed, and resuspended in medium with SB202190 (10 μM)or vehicle control (0.1% DMSO) for 120 min. Rh123 fluorescence was measured using FAC scan. Means ± SD from three independent experiments. *P < 0.05 vs SGC7901 cells. **P < 0.05 vs vehicle control.

Our previous data clearly showed that the p38-MAPK/AP-1 pathway was activated in SGC7901/VCR cells. Moreover, AP-1 was reported to be involved in *MDR1 *upregulation [11]. We then investigated the effects of p38-MAPK inhibition on *MDR1 *expression and function of P-gp. Western-blot analysis showed that inhibition of p38-MAPK by SB202190 markedly downregulated P-gp expression in SGC7901/VCR cells. This effect was confirmed by RT-PCR analysis (Fig. 3A–C). To examine the effect of SB202190 on function of P-gp, Rh123 accumulation and efflux studies were chosen. As shown in Figure 3D, Rh123 accumulation and retention in SGC7901/VCR cells were obviously less than that in SGC7901 cells. After treatment with SB202190 (10 μM), Rh123 accumulation and retention in SGC7901/VCR cells were increased; however, there was no change in SGC7901 cells(Fig. 3D). Thus, our results clearly showed that inhibition of p38-MAPK reduced *MDR1 *gene expression and function of P-gp in SGC7901/VCR cells.

**Figure 4:**
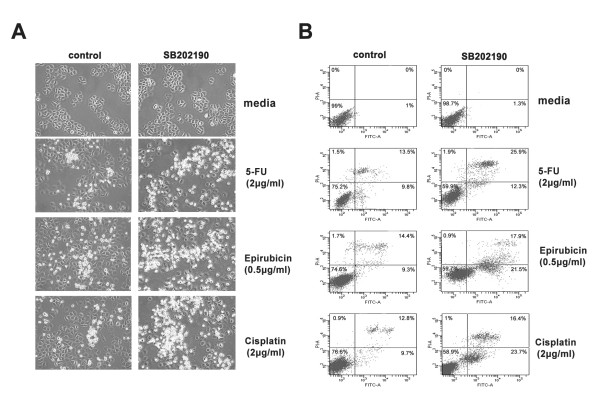
**Inhibition of p38 MAPK increases SGC7901/VCR to chemotherapy**. SGC7901/VCR cells were treated with indicated concentrations of cisplatin, 5-FU or epirubicin, with or without SB202190 (10 μM), for 24 hours. (A) The morphology of cells was observed, and photographs were taken under the microscope. (B) Flow cytometry analysis was performed after staining with Annexin V/PI. Compared with the control cells (without SB202190), there were significantly more apoptotic cells in the SB230920 treatment group. A representative example of an experiment that was repeated three times is shown.

Upregulation of the *MDR1 *gene has been reported to be closely related to chemotherapy resistance, and inhibition of the *MDR1 *gene has been shown to increase the sensitivity of tumor cells to P-gp-related chemotherapeutic agents [30]. Although cisplatin and 5-FU are not the substrates of P-gp, it has been reported that increased expression of c-jun contributed to cisplatin resistance, meanwhile it was also reported that 5-FU can induce c-jun phosphorylation and activate both AP-1-specific transcription and DNA binding [31,32]. We therefore tested whether inhibition of p38-MAPK could restore the chemotherapeutic sensitivity of drug-resistant tumor cells to P-gp-related drug and P-gp-unrelated drugs. SGC7901/VCR cells were treated with 5-FU, cisplatin and eprubicin for 24 hours, with or without 2-hour pretreatment with SB202190 (10 μM). The morphology of cells was observed, and photographs were taken under the microscope. Annexin-V/PI staining analysis was performed to detect cell apoptosis. As shown in Fig. 4A, cells treated with SB202190, but not control cells (not treated with SB202190), showed significantly increased levels of cell death and exhibited typical morphologic features of apoptotic cell death, including cytoplasmic condensation, nuclear fragmentation and membrane blebbing. These results were confirmed by Annexin-V/PI staining analysis (Fig. 4B): cells treated with SB202190 showed significantly increased levels of apoptosis. Taken together, these results indicate that inhibition of p38-MAPK increases the sensitivity of SGC7901/VCR cells to chemotherapy

## Discussion

Multidrug resistance (MDR) is a phenomenon by which tumor cells develop cross-resistance to a range of structurally and functionally unrelated drugs [33]. MDR is one of the main obstacles to successful cancer therapy [34]. Many different mechanisms have been suggested to explain the development of a MDR phenotype in cancer cells, one of the extensively studied form of these mechanisms is overexpression of several energy dependent drug efflux pumps that belong to the ATP-binding cassette family of transporters, such as the P-glycoprotein (P-gp) and the MDR-associated proteins (MRPs) [33]. P-gp is a product of the *MDR1 *gene. Overexpression of *MDR1 *has often been reported in primary gastric adenocarcinoma [35,36]. However, there are some reports showing that P-gp-associated MDR cells develop other mechanisms to acquire chemoresistance to P-gp-unrelated drugs [5-9]. In this study, we found that constitutive overexpression of the *MDR1 *gene in drug-resistant human gastric cancer SGC7901/VCR cells is dependent on phosphorylation of p38 and the activity of AP-1. Inhibition of p38-MAPK could restore the sensitivity of these cells to P-gp-related drug and P-gp-unrelated drugs.

Cultured tumor cells, when selected for resistance to an anti-neoplastic agent, often acquire cross-resistance to others. In this study, we showed that vincristine-rsistant SGC7901/VCR is a multidrug-resistant cancer cell line, which was resistant to P-gp-related drug (eprubicin) and P-gp-unrelated drugs (5-FU and cisplatin). Vincristine and epirubicin are known substrates of P-glycoprotein, and it is now increasingly evident that P-gp-associated MDR cells were conferred a cross-resistance to P-gp-unrelated drugs [8,9]. We then examined and compared the levels of *MDR1 *gene expression in SGC7901/VCR and SGC7901 cells, and showed SGC7901/VCR cells have increased expression levels of P-gp protein and MDR1 mRNA than SGC7901. But cisplatin or 5-FU treatment could not induce MDR1 gene expression in SGC7901 cells. Taken together, these results suggested SGC7901/VCR cells may develop P-gp-related or unrelated mechanism to acquire chemoresistance. Thus, P-gp-associated MDR cell line-SGC7901/VCR has ability to crossresist against P-gp-related drug (eprubicin) and P-gp-unrelated drugs (5-FU and cisplatin). Our results was in agreement with previous findings that the P-gp-prominent MDR cell was cross-resistant to 5-FU and cisplatin[37].

It has previously been reported that the human *MDR1 *promoter contains an AP-1-binding site [38], and increased AP-1 binding [39] has been observed in several multidrug-resistant cell lines whereas reduced AP-1 binding has been associated with increased drug sensitivity in others [11,40]. In this study, we observed increased AP-1 activity in SGC7901/VCR cells compared with the drug-sensitive parental SGC7901 cells. This result indicates that increased activity of AP-1 is correlated with increased drug resistance in SGC7901/VCR cells.

Recently, increasing evidence indicates that anticancer drugs activate many signal pathways, some of which are connected to the development of drug resistance of tumor cells [41]. The MAP kinase pathway is an important signal-transduction pathway activated by many different stimuli. Previous reports have shown that modulators of the MAP kinase pathway can affect drug transport activity of P-gp in certain multidrug-resistant cell lines [42,43]. We investigated the role of the MAP kinase signal pathway in drug-resistant and drug-sensitive cell lines. As shown in Fig. 3B and 3C, phosphorylation of p38 was increased in SGC7901/VCR cells, but there were no differences in phosphorylation of JNKs and ERKs between drug-resistant and drug-sensitive cell lines. Meanwhile, AP-1 activity was significant attenuated by SB202190 or after cotransfection with the DN-p38 plasmid. We also observed that inhibition of p38 by SB202190 markedly decreased levels of P-gp, MDR1 mRNA and function of P-gp. Moreover, the function of P-gp can also be repressed by SB202190 (Fig. 3D) in SGC7901/VCR cells. Furthermore, incubation with cisplatin for 24 h can also induce p38 phosphorylation in SGC7901 cells; this result is supported by previous report that p38 MAPK was preferentially activated by cisplatin in several cell lines [44]. Thus, these results suggest that the p38-MAPK/AP-1 signal pathway is involved in regulation of the *MDR1 *gene in SGC7901/VCR cells, and may contribute to chemoresistantce. Our results were in line with previous report that the specific p38-MAPK inhibitor SB203580 blocked both c-fos and c-jun expression in response to UV irradiation and anisomycin [45]. Although p38-MAPK does not phosphorylate or activate c-Jun, several lines of evidence however, support the idea that p38 can contribute to AP-1 activity. p38 can contribute to c-jun gene induction mediated by the AP-1 binding site in the c-jun promoter. In addition to the AP-1 site, the c-jun gene can be regulated by MEF2 family of transcription factors (consisting of MEF2 A-D) [46]. The MEF2 site is critical for induction of the c-jun promoter by LPS in macrophages, and that this induction requires functional p38 and transcription factor MEF2C. Furthermore, MEF2C was shown to be directly phosphorylated and activated by p38 [47]. Thus, p38 can potentially regulate c-Jun transcriptional activity by regulating transcription factors that bind to the AP-1 site as well as the MEF2 site.

It has been previously reported in other cell lines that p38-MAPK does not affect P-gp and MDR1 mRNA expression [48,49]. In this study, we demonstrated that inhibition of p38-MAPK by SB202190 significantly attenuated the activity of AP-1 and *MDR1 *gene expression. As AP-1 is the important transcription factor for *MDR1 *gene expression, we suggest that SB202190 inhibited *MDR1 *gene expression by reducing the activity of transcription factor AP-1. Thus, the effect of p38-MAPK on *MDR1 *gene expression is cell type dependent.

Previous reports have showed that increased expression of *c*-*Jun *contributed to cisplatin resistance, and 5-FU can induce *c-Jun *phosphorylation and activate AP-1-specific transcription [31,32]. Meanwhile, we have demonstrated that the activity of Ap-1 was upregulated in SGC7901/VCR cells and inhibition of p38-MAPK by SB202190 can reduce the activity of AP-1 and inhibit *MDR1 *gene expression. Therefore, we investigated the effect of SB202190 on the multidrug-resistant phenotype of drug-resistant SGC7901/VCR cells. As shown in Fig. 6, SB202190 significantly increased the sensitivity of drug-resistant SGC7901/VCR cells to chemotherapeutic agents, which indicates that SB202190 may reverse the multidrug-resistant phenotype in SGC7901/VCR cells. Our results were consistent with previous reports that pharmaceutical inhibition of p38 by SB203580 reversed the multidrug resistance of L1210/VCR cells [42]. It has also been reported that the p38 MAPK pathways play an important role in cellular resistance against photodynamic therapy with hypericin in HeLa cells [50]. Therefore, it could be suggested from our results that drug resistance in SGC7901/VCR cells is attributed at least in part to the activation of p38-MAPK/AP-1 signal pathway, and downregulation of this pathway appears to confer on MDR cells sensitivity to P-gp-unrelated drugs as well as P-gp-related drug. Several reports have showed that specific inhibitors of signal pathway can reverse P-gp-mediated multidrug resistance [42,43,51,52]. Thus, interrupting signal-transduction pathways that mediate the expression of multidrug transporters might be an effective approach to prevent multidrug resistance and increase sensitivity to chemotherapy in human cancers. Taken together, our data indicate that the p38-MAPK signal pathway affects *MDR1 *gene expression, and provide a possible new mechanism for cross-resistance in drug-resistant human gastric cancer cells. These results further our understanding of the regulatory mechanisms involved in *MDR1 *gene expression and may provide new strategies for reversal of multidrug resistance in human gastric cancer cells.

## Conclusion

Activation of the p38-MAPK pathway might be responsible for the modulation of P-glycoprotein-mediated multidrug resistance in the SGC7901/VCR cell line.

## Abbreviations

MAPK: mitogen-activated protein kinases; MDR: multidrug resistance; AP-1: activator protein-1.

## Competing interests

The authors declare that they have no competing interests.

## Authors' contributions

XLG and NNM carried out the molecular genetic studies, participated in the sequence alignment and drafted the manuscript. JW, JRS and XXB carried out the immunoassays. YC participated in the sequence alignment. KS and HYX carried out cellular studies. GCJ and BHZ participated in the design of the study and performed the statistical analysis. MCW and LXW conceived of the study, and participated in its design and coordination. All authors read and approved the final manuscript.

## Pre-publication history

The pre-publication history for this paper can be accessed here:



## Supplementary Material

Additional file 1 Cytotoxicity of different chemotherapeutic drugs in SGC7901 and SGC7901/VCR cell lines expressed as IC_50 _* values obtained by MTT assay. The data provided represent the statistical analysis of the cytotoxicity of different chemotherapeutic drugs in SGC7901 and SGC7901/VCR cell lines expressed as IC_50 _* values, *Each IC_50 _value (lethal dosage required to inhibit 50% of cell growth) is the average IC_50 _value of three independent MTT assay. Relative resistance is defined as the IC_50 _value of the drug-resistant cells divided by the IC_50 _value of the parent SGC7901 cells (concentrations are expressed in μg/ml). Click here for file
