# Supplementary material for: Increased p38-MAPK is responsible for chemotherapy resistance in human gastric cancer cells
Source: BMC Cancer. 2008 Dec 18;8:375. doi: 10.1186/1471-2407-8-375 (PMC2628930; doi:10.1186/1471-2407-8-375)
Supplement: Additional file 1 — Cytotoxicity of different chemotherapeutic drugs in SGC7901 and SGC7901/VCR cell lines expressed as IC50 * values obtained by MTT assay. The data provided represent the statistical analysis of the cytotoxicity of different chemotherapeutic drugs in SGC7901 and SGC7901/VCR cell lines expressed as IC50 * values, *Each IC50 value (lethal dosage required to inhibit 50% of cell growth) is the average IC50 value of three independent MTT assay. Relative resistance is defined as the IC50 value of the drug-resistant cells divided by the IC50 value of the parent SGC7901 cells (concentrations are expressed in μg/ml). [file 1471-2407-8-375-S1.doc]

Table 1

Cytotoxicity of different chemotherapeutic drugs in SGC7901 and SGC7901/VCR cell lines expressed as IC50* values obtained by MTT assay

| Chemotherapeutic drugs SGC7901 SGC7901/VCR Relative resistance  for SGC7901/VCR |
| --- |
| 5-Fluorouracil 0.92 ± 0.02 6.2 ± 0.05 6.74  Cisplatin 0.49 ± 0.06 2.9 ± 0.017 5.92  Epirubicin 0.24 ± 0.0026 0.82 ±0.003 3.42 |

*Each IC50 value (lethal dosage required to inhibit 50% of cell growth) is the average IC50 value of three independent MTT assay. Relative resistance is defined as the IC50 value of the drug-resistant cells divided by the IC50 value of the parent SGC7901 cells (concentrations are expressed in μg/ml).
